# Sevoflurane Inhibits the Th2 Response and NLRP3 Expression in Murine Allergic Airway Inflammation

**DOI:** 10.1155/2018/9021037

**Published:** 2018-09-30

**Authors:** Lixia Wang, Binshan Zha, Qiying Shen, Hongyun Zou, Cheng Cheng, Huimei Wu, Rongyu Liu

**Affiliations:** ^1^Department of Pulmonary, Anhui Geriatric Institute, The First Affiliated Hospital of Anhui Medical University, Jixi Road 218, Hefei, Anhui 230022, China; ^2^Department of Anesthesiology, The First Affiliated Hospital of Anhui Medical University, Jixi Road 218, Hefei, Anhui 230022, China; ^3^Department of Vascular Surgery, The First Affiliated Hospital of Anhui Medical University, Jixi Road 218, Hefei, Anhui 230022, China

## Abstract

**Background:**

Our colleagues have demonstrated an impressive therapeutic role of sevoflurane in a murine allergic airway inflammation model, but the mechanisms underlying this effect remain undefined. In this study, we tried to investigate the effect of sevoflurane on the resolution of allergic airway inflammation and to assess whether NLRP3 or the NLRP3 inflammasome is involved in this process.

**Methods:**

Female (C57BL/6) mice were sensitized and challenged with ovalbumin (OVA). Then, some of the mice received MCC950 (10 mg/kg; i.p.) or 3% sevoflurane. Total and differential inflammatory cell numbers, proinflammatory cytokines in bronchoalveolar lavage fluid (BALF), the peribronchial inflammation density, and mucus production were evaluated. In addition, we analysed the protein levels of NLRP3, the apoptosis-associated speck-like protein containing the caspase activation and recruitment domain (ASC), pro-caspase-1, and caspase-1 in the lung tissue.

**Results:**

We found that OVA-induced inflammatory cell recruitment to peribronchial regions, goblet cell hyperplasia, the serum levels of IgE, inflammatory cells, and the Th2 cytokine secretion in BALF was potently suppressed by sevoflurane with an efficacy comparable with that suppressed by MCC950 treatment. Furthermore, sevoflurane, similar to MCC950, clearly inhibited the OVA-induced activity of NLRP3 in the lungs. In addition, we found that OVA challenge failed to increase the expression of ASC, pro-caspase-1, and caspase-1 in the lungs and the levels of IL-18 and IL-1*β* in BALF.

**Conclusion:**

Taken together, our data showed that sevoflurane ameliorated allergic airway inflammation by inhibiting Th2 responses and NLRP3 expression. The NLRP3 independent of inflammasomes participated in the pathogenesis of allergic asthma in this model.

## 1. Introduction

Allergic airway inflammation is a chronic inflammatory disease of the airways characterized by T-helper 2- (Th2-) mediated immune responses to common aeroallergens in genetically susceptible individuals [1, 2]. Each reexposure to allergen results in type E immunoglobulin (IgE) production and Th2 cell activation [3]. Upon sustained activation, Th2 cells produce proinflammatory cytokines that play pivotal roles in the amplification of inflammatory processes [4]. In addition, persistent inflammation leads to excessive secretion of mucus, hyperplasia/hypertrophy of smooth muscle, and airway remodelling [5]. Therefore, the ideal therapeutic approach for allergic airway disease is to achieve inflammatory control.

Nucleotide-binding domain and leucine-rich repeat protein 3 (NLRP3) is one of the most-studied members of the NLR family of receptors. The most well-studied role of NLRP3 involves the formation of the NLRP3 inflammasome. The NLRP3 inflammasome is composed of NLRP3, the apoptosis-associated speck-like protein containing the caspase activation and recruitment domain (ASC), and caspase-1 [6]. The assembly of the above three components activates caspase-1, which in turn results in the cleavage of pro-IL-1*β* and pro-IL-18 into their mature forms [7]. Recently, the immunological function of NLRP3 independently of the inflammasome was reported. Bruchard and colleagues demonstrated that NLRP3 expression in CD4+ T cells specifically supported Th2 transcription in a cell-intrinsic manner, and the ability of NLRP3 to control Th2 polarization was involved in the promotion of asthma, independent of inflammasome activation [8].

Sevoflurane, a commonly used volatile anaesthetic, has been used as a last-resort treatment for life-threatening asthma in children [9]. Apart from bronchodilation [10–12], our coworkers have confirmed that sevoflurane suppressed allergic airway inflammation by inhibiting inflammatory infiltrates and mucus production, as well as keeping balance of cytokine responses [13]. However, whether sevoflurane inhibits the Th2 response and NLRP3 inflammasome activation in allergic airway inflammation remains unknown.

In the present study, we investigated whether sevoflurane inhibits the activation of the NLRP3 inflammasome to mitigate allergic airway inflammation. In addition, the impact of sevoflurane on the Th1 and Th2 responses was also investigated.

## 2. Methods

### 2.1. Mice

Female C57BL/6 mice, aged 6 to 7 weeks, were obtained from the Shanghai Laboratory Animal Centre (Shanghai, China). The mice were housed under a 12 h light/dark cycle at an ambient temperature of 24 ± 1°C in a specific pathogen-free animal facility. All the experiments with mice were performed according to protocols approved by the Committee on the Ethics of Animal Care and Use of Anhui Medical University.

### 2.2. Induction of Allergic Airway Inflammation and Treatments

Thirty mice were assigned randomly into five groups (*n* = 6 each): (1) phosphate-buffered saline control (Con); (2) MCC950 control (MCC); (3) ovalbumin- (OVA-) induced lung allergic inflammatory group (OVA); (4) OVA group treated with sevoflurane (OVA + SVF); and (5) OVA group treated with MCC950 (OVA + MCC).

OVA sensitization was performed by intraperitoneal (i.p.) injection of 10 *μ*g of OVA (Sigma, St. Louis, MO, USA) adsorbed to 1 mg of alum (potassium aluminium sulphate, Sangon Biotech, Shanghai, China) on day 0. Fourteen days later, the animals were challenged by 1% OVA solution for 30 min for 7 consecutive days. The control mice were sensitized and challenged with saline alone. Immediately after OVA provocation, the mice received 3% sevoflurane (Maruishi Pharmaceutical, Osaka, Japan) for 1 h from day 14 to 20. MCC950 (10 mg/kg, i.p.) (Abmole Bioscience Inc., USA), a recently-described small molecule inhibitor of the NLRP3 inflammasome, was administered every two days postchallenge with OVA for comparison. At 24 h after the final challenge, the mice were sacrificed, and bronchoalveolar lavage fluid (BALF) was collected. Half of each lung was stored at −80°C for Western blotting, and the other half was fixed overnight in buffered 4% formaldehyde solution for histology analysis.

### 2.3. Bronchoalveolar Lavage and Differential Cell Counts

Immediately after sacrifice, the BALF from the left lung was harvested through an endotracheal tube. The left lungs were washed three times with 0.5 mL of saline solution, and the BALF was collected with an average fluid recovery rate of greater than 90%. Then, the BALF was centrifuged at 700 *g* for 5 min at 4°C, and the supernatant was separated and stored at −80°C. The pellet was resuspended and subjected to differential cell counting after Wright-Giemsa staining. The total cells were counted using a haemocytometer.

### 2.4. Enzyme-Linked Immunosorbent Assay (ELISA)

Commercial ELISA kits (Cusabio, Wuhan, China) were used to measure OVA-specific IgE levels in the serum. In addition, the production of IL-4, IL-13, IFN-*γ*, IL-18, and IL-1*β* in the BALF was examined using an ELISA purchased from Cusabio (Wuhan, China) according to the protocols provided by the manufacturer. The detection limits for IL-4, IL-13, IFN-*γ*, IL-18, and IL-1*β* were 1.56 pg/mL, 31.25 pg/mL, 15.6 pg/mL, 1.56 pg/mL, and 1.56 pg/mL, respectively.

### 2.5. Lung Histological Assessment

The right lung of each mouse was dissected free, fixed in 4% formaldehyde, and embedded in paraffin. Then, the paraffin-embedded tissue sections (5 *μ*m) were stained with haematoxylin/eosin (H&E) or periodic acid-Schiff (PAS). For the quantification of inflammatory cells in H&E-stained lung sections, a semiquantitative scoring system described by Koltsida et al. was used [14]. In addition, goblet cell hyperplasia quantification was accomplished by dividing the number of PAS^+^ cells in the airway by the perimeter of the basement membrane (Pbm). At least 4 different fields of view per animal were evaluated.

### 2.6. Western Blot

Lung tissue was homogenized in RIPA buffer with a protease inhibitor (Roche, Indianapolis, IN, USA) and the phosphatase inhibitor PhosSTOP (Roche). Total protein concentration was measured using the microbicinchoninic acid assay, in which 25 *μ*g of total protein per lane was separated on 12% SDS-PAGE gels. Then, the proteins were transferred to PVDF membranes (Millipore, Billerica, MA, USA) and blocked with 5% nonfat milk for 1 h at room temperature. Thereafter, the membranes were probed with antibodies against GAPDH (KANGCHEN Biotech, Shanghai, China), NLRP3 (Signaling Technology Inc., Beverly, MA, USA), ASC (Signaling Technology Inc.), and caspase-1 (Abcam, Boston, MA, USA). ImageJ software was used to quantify protein bands.

### 2.7. Statistical Methods

All the data were expressed as the mean ± SEM, and statistical analysis was performed with SPSS19. ANOVA was used to compare differences between multiple groups, followed by the post hoc Bonferroni's test for selected pairs. *P* < 0.05 was considered significant.

## 3. Results

### 3.1. Sevoflurane and MCC950 Ameliorate Established OVA-Induced Pathologic Features

**Figure 1:**
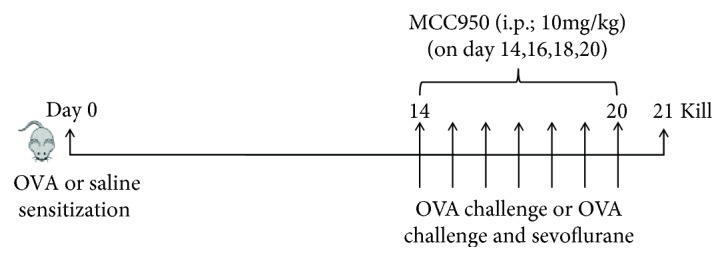
Schematic diagram showing the workflow of the experiment. Mice were sensitized by intraperitoneal (i.p.) injection of OVA or saline on day 0. Then, aerosolized OVA or saline solution was administered on days 14–20 for 30 min per day. After OVA challenge, some mice received 3% sevoflurane for 1 h on day 14–20 or MCC950 (10 mg/kg, i.p.) every second day.

**Figure 2:**
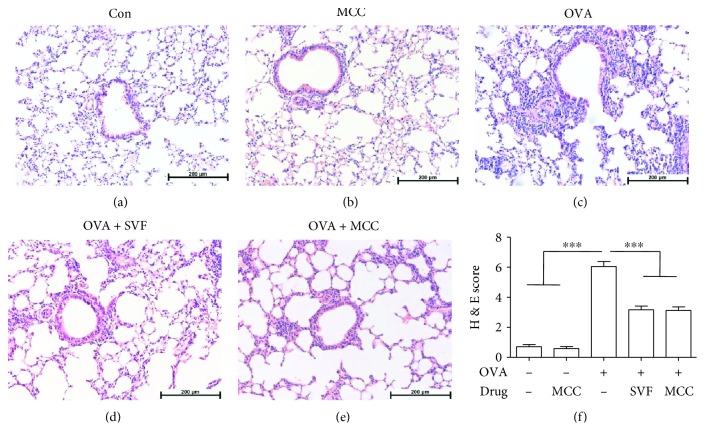
Effect of sevoflurane and MCC950 on the pathologic features of OVA-induced allergic airway inflammation. (a–e) Representative H&E-stained sections of lungs from different groups (magnification ×200). (f) Histological scoring was expressed as the mean ± SEM of 6 mice per group from three independent experiments, ^∗∗∗^*P* < 0.001. Peribronchial inflammation was scored with a maximum score of 8 as follows: 0, normal; 1, few cells; 2, a ring of inflammatory cells one cell layer deep; 3, a ring of inflammatory cells two to four cells deep; 4, a ring of inflammatory cells of more than four cells deep.

To investigate the therapeutic potential of sevoflurane in reducing airway inflammation in allergic asthma, the mice were sensitized and challenged with OVA, as illustrated in Figure 1. We then evaluated inflammatory cell infiltration of the area surrounding the airways in the lung by histological analysis. OVA challenge significantly increased the inflammatory cells concentrated near the airways (Figure 2(c)), while sevoflurane or MCC950 treatment resulted in a marked decrease in inflammatory cell infiltration (Figures 2(d)–2(f)).

### 3.2. Sevoflurane and MCC950 Decrease Allergen-Induced Leukocyte Recruitment into the Lung

**Figure 3:**
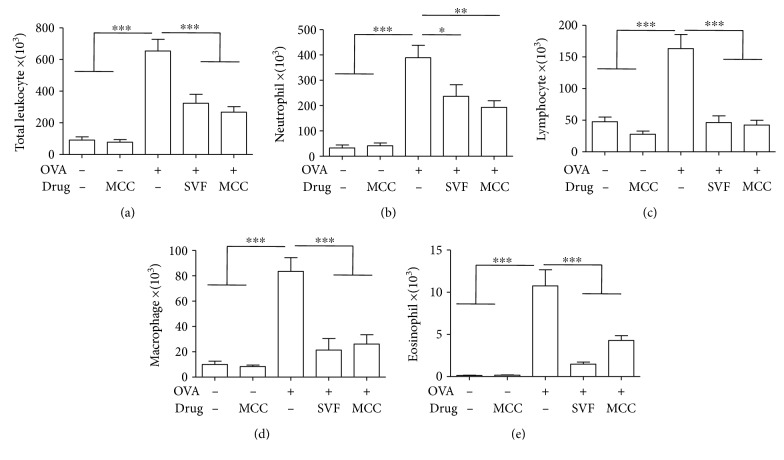
Effect of sevoflurane and MCC950 on OVA-induced inflammatory cells in the BALF. Data shown represent changes in the total number of cells (a), neutrophils (b), lymphocytes (c), macrophages (d) and eosinophils (e) in the BALF. Data are expressed as the mean ± SEM of six mice per group, ^∗^*P* < 0.05, ^∗∗^*P* < 0.01, ^∗∗∗^*P* < 0.001.

The onset of asthma is characterized by the influx of inflammatory cells, including eosinophils and neutrophils, into the lung. Compared with control mice, there was a dramatic increase in the total leucocyte numbers detected in the BALF of allergic mice (Figure 3(a)). Such an increase in leucocyte counts included elevated numbers of neutrophils (Figure 3(b)), lymphocytes (Figure 3(c)), macrophages (Figure 3(d)), and eosinophils (Figure 3(e)). However, treatment with sevoflurane or MCC950 produced a profound reduction in the total numbers and differential counts of inflammatory cells in BALF.

### 3.3. Sevoflurane and MCC950 Significantly Reduce Goblet Cell Hyperplasia in Allergic Mice

**Figure 4:**
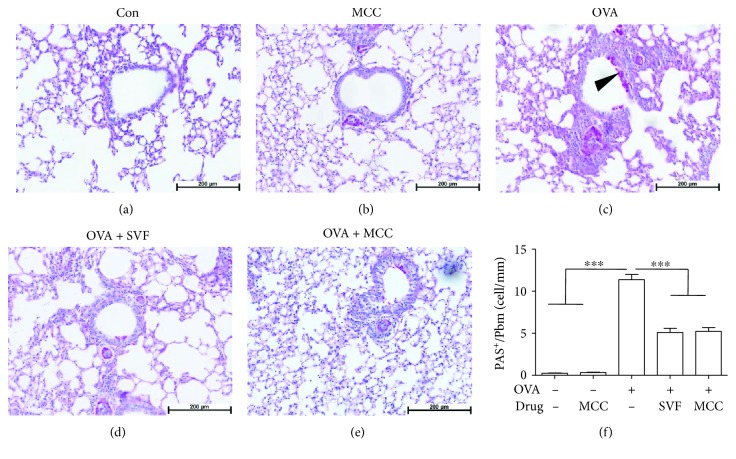
Effect of sevoflurane and MCC950 on allergen-induced mucus production in the lung. Representative periodic acid-Schiff (PAS) staining of lung sections from mice challenged with saline (a), MCC950 (b), OVA (c), OVA+ SVF (d) and OVA+MCC950 (e). (f), Quantification of PAS^+^ cells in the airway. Data are expressed as the mean ± SEM, ^∗∗∗^*P* < 0.001, *n* = 6 for each group.

After OVA challenge, mucus secretion and PAS^+^ cells surrounding the airway were markedly increased compared with those in the control group. However, sevoflurane or MCC950 treatment resulted in a marked reduction in goblet cell hyperplasia (Figures 4(a)–4(f)).

### 3.4. Sevoflurane and MCC950 Cause a Decrease in the Levels of Serum IgE and Th2 Cytokine and Restore the Th1/Th2 Balance

**Figure 5:**
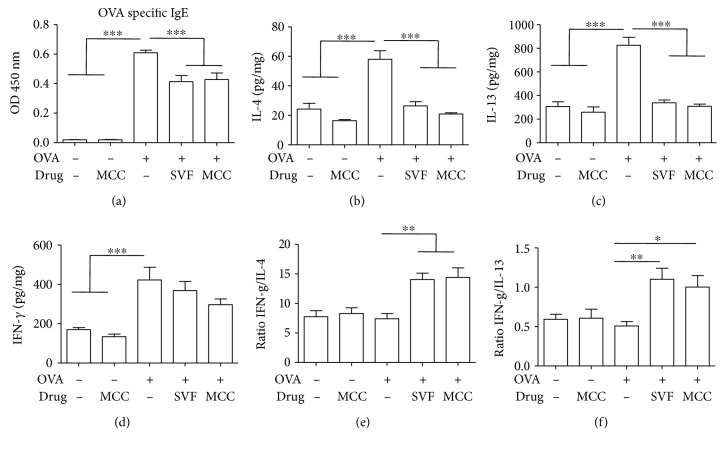
Effect of sevoflurane and MCC950 on IgE levels in serum and Th1 and Th2 cytokine levels in the BALF. (a) OVA-specific IgE levels were measured in serum samples. (b–d) Production of IL-4, IL-13 and IFN-*γ* in the BALF. (e) The ratio of IFN-*γ* to IL-4. (f) The ratio of IFN-*γ* to IL-13. Data are expressed as the mean ± SEM of six mice per group, ^∗^*P* < 0.05, ^∗∗^*P* < 0.01, ^∗∗∗^*P* < 0.001.

The increase in serum OVA-specific IgE is generally considered the hallmark of asthma onset [3]. As expected, OVA challenge significantly increased the production of serum IgE compared with that of the control groups. Sevoflurane or MCC950 treatment significantly decreased OVA-induced IgE levels, although these levels remained higher than those in the control mice (Figure 5(a)).

The IgE-mediated immune response in asthma is closely related to increased Th2 cytokine levels [3]. Additionally, maintaining a balance between Th2 and Th1 cytokines is a key therapeutic approach for asthma [15, 16]. Therefore, the production of both Th1 and Th2 cytokines in BALF were detected by ELISA.

After OVA challenge, both the Th2 cytokines IL-4 (Figure 5(b)) and IL-13 (Figure 5(c)) and the Th1 cytokine IFN-*γ* (Figure 5(d)) were significantly increased compared with those of the control animals. However, sevoflurane or MCC950 treatment led to a dramatic decrease in Th2 cytokine production elicited by OVA challenge, with no difference in Th1 cytokine levels (Figures 5(b)–5(d)). Furthermore, the ratios of Th1 to Th2 cytokines in BALF from the sevoflurane or MCC950 treatment group of mice were significantly higher than those in the other groups (Figures 5(e) and 5(f)).

### 3.5. Sevoflurane Reduces NLRP3 Protein Expression

**Figure 6:**
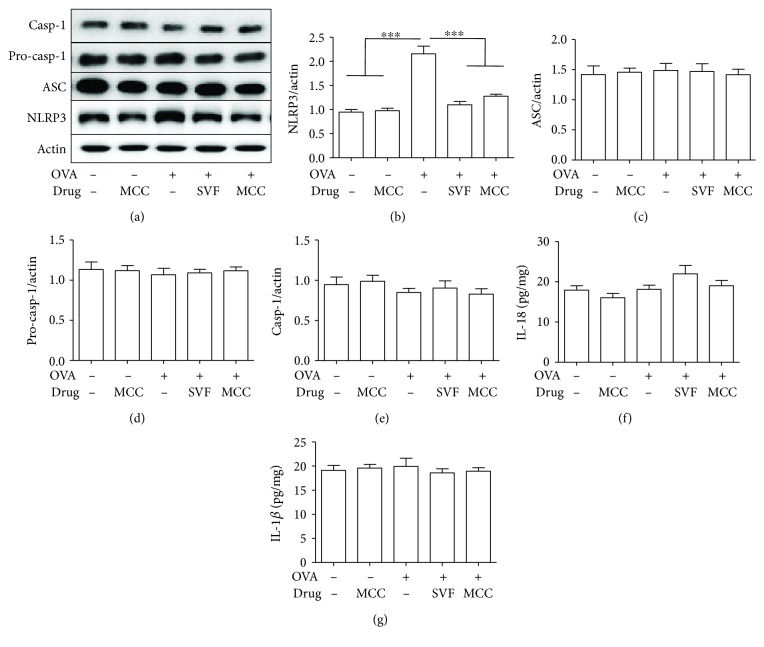
Effect of sevoflurane and MCC950 on NLRP3, ASC, pro-caspase-1 and caspase-1 expression in lung tissue. (a) Western blot analysis of NLRP3, ASC, pro-caspase-1, caspase-1 and actin expression in lung tissue. (b–e) Relative levels of NLRP3, ASC, pro-caspase-1 and caspase-1 expression in lung tissue. (f, g) Production of IL-18 and IL-1*β* in the BALF. Data are expressed as the mean ± SEM of six mice per group from three separate experiments, ^∗∗∗^*P* < 0.001.

Several studies have revealed that NLRP3 inflammasome activation is involved in the pathogenesis of asthma [17–20]. First, we hypothesized that NLRP3 may direct inflammasome activation in asthma. Since the activation of inflammasomes relies on the assembly of not only NLRP3 but also ASC and caspase-1, we used Western blot analysis to assess the expression of these proteins in the lung. As illustrated in Figure 6, OVA challenge substantially increased NLRP3 protein expression but failed to increase the expression of ASC or caspase-1. Considering that inflammasome activation results in the secretion of mature IL-1*β* and IL-18, we further assessed the levels of IL-18 and IL-1*β* in BALF. However, we found that neither IL-18 nor IL-1*β* in BALF was increased in allergic mice. More interestingly, we found that sevoflurane abolished the OVA-induced expression of NLRP3 (Figure 3(b)), which is similar to that observed in MCC950-treated allergic mice. These results suggested that NLRP3 mediated allergic airway inflammation independently of inflammasome activation, while sevoflurane inhibited the activity of NLRP3 to alleviate airway inflammation.

## 4. Discussion

In this study, we found that sevoflurane suppressed inflammatory cell recruitment to peribronchial regions and inhibited goblet cell hyperplasia. Sevoflurane therapy was also associated with inhibition of inflammatory cell invasion into BALF and reduced serum levels of IgE and OVA-induced Th2 cytokines in BALF. Furthermore, sevoflurane inhibited the OVA-induced activity of NLRP3 in the lungs, which is similar to that of MCC950-treated allergic mice. In addition, we found that the activation of NLRP3 helped promote allergic asthma and that this process was independent of inflammasome activation.

During the last decade, both clinical and experimental studies have shown the beneficial effect of sevoflurane on asthma by reducing airway resistance [9, 11, 21], although little is known regarding the exact mechanisms. The proposed mechanisms include a reduction in vagal tone, direct relaxation of smooth muscle tissue, inhibition of the release of bronchoconstrictive mediators, and synergy with catecholamines [22, 23]. However, we cannot ignore the finding that airway inflammation contributes to airway resistance in asthmatics [24]. During acute episodes of asthma, airway resistance may be associated with IgE-mediated bronchoconstriction, and prolonged or nonresolving inflammation results in airway remodelling that can aggravate airway resistance [25]. Therefore, it is reasonable to speculate that sevoflurane reduces airway resistance by inhibiting airway inflammation. Previous studies have demonstrated that sevoflurane attenuated oleic acid-induced pulmonary oedema, decreased tumour nuclear factor-*α*, and ameliorated the alveolar damage score in dogs; sevoflurane was also shown to benefit patients with acute lung injury [26, 27]. The results of previous studies in our laboratory show that the OVA-mediated increases in inflammatory cells around bronchi, goblet cell hyperplasia, and total numbers and differential counts of inflammatory cells in BALF were significantly inhibited by sevoflurane [13]. In accordance with the results of our colleagues, this study confirms the anti-inflammatory effects of sevoflurane, which is similar to MCC950.

Allergic asthma is known to result from immunologic reactions, and the Th2 immune pathway plays a key role in the initiation and perpetuation of airway inflammation [28]. Persistent Th2 immune responses produce abundant Th2 cytokines, of which IL-4 plays a key role in inducing IgE production in B lymphocytes [29]. Another important Th2 cytokine, IL-13, contributes to goblet cell hyperplasia [30]. In this study, we found that OVA-induced IL-4 and IL-13 levels in BALF was counteracted by sevoflurane with an efficacy comparable with that counteracted by MCC950 treatment. Although sevoflurane and MCC950 failed to reduce the level of IFN-*γ*, a Th1-type cytokine, the ratio of Th1 to Th2 cytokines was substantially increased. These results suggest that sevoflurane and MCC950 may exert its effect by inhibiting Th2 responses and by achieving a balance between Th2 and Th1 cytokines. Our results support the notion that maintaining a balance between the Th2 and Th1 responses may be a key therapeutic approach for allergic airway inflammation [31].

Sevoflurane clearly ameliorated allergic airway inflammation by inhibiting Th2 responses, although the mechanism involved was less clear. In asthma models, the role of the NLRP3 inflammasome remains controversial. The NLRP3 inflammasome was reported to be involved in the inflammatory processes of airway diseases such as asthma [17, 18, 32, 33], while the NLRP3 inflammasome failed to show a functional role in asthma models [8, 34]. In our work, OVA challenge substantially increased the protein expression of NLRP3, although the protein levels of pro-caspase-1, caspase-1, and ASC and the levels of IL-18 and IL-1*β* in BALF were not altered. Moreover, inhibition of NLRP3 by MCC950 significantly inhibited allergic airway inflammation. Therefore, our results suggested that the activation of NLRP3 promoted asthma, and its effect was unlikely to rely on NLRP3 inflammasome activation. Surprisingly, we observed that sevoflurane, similar to MCC950, significantly decreased OVA-induced expression of NLRP3. Altogether, these observations suggest the inhibitory effects of sevoflurane on Th2 cytokine and NLRP3 expression. Recently, it has been reported that NLRP3 acts as a key transcription factor in Th2 differentiation in asthma models [8], suggesting that sevoflurane may inhibit Th2 responses via NLRP3 in our model.

In conclusion, our study suggests that the protective effect of sevoflurane on allergic airway inflammation is at least partly explained by the inhibition of Th2 cytokines and NLRP3 expression. The NLRP3 independent of inflammasomes participated in the pathogenesis of allergic asthma in this model.

## Data Availability

The data used to support the findings of this study are included within the article. And the data used to support the findings of this study are available from the corresponding author upon request.

## Abbreviations

NLRP3: Nucleotide-binding domain and leucine-rich repeat protein 3
OVA: Ovalbumin
BALF: Bronchoalveolar lavage fluid
NLR: NOD-like receptor
ASC: Apoptosis-associated speck-like protein containing a CARD
IgE: Immunoglobulin E.
